# Prognostic Value of Carbonic Anhydrase IX Immunohistochemical Expression in Renal Cell Carcinoma: A Meta-Analysis of the Literature

**DOI:** 10.1371/journal.pone.0114096

**Published:** 2014-11-26

**Authors:** Zhihong Zhao, Guixiang Liao, Yongqiang Li, Shulu Zhou, Hequn Zou, Samitha Fernando

**Affiliations:** 1 Institution of Urology and Nephrology, The third Affiliated Hospital of Southern Medical University, Guangzhou, China; 2 Department of Radiation Oncology, Nanfang Hospital, Southern Medical University, Guangzhou, China; 3 Imperial College London, London, United Kingdom; University Health Network, Canada

## Abstract

**Background:**

Carbonic anhydrase IX (CAIX) protein has been correlated with progression and survival in patients with renal cell carcinoma (RCC). The prognostic value of CAIX in RCC however, remains inconclusive according to published works. This study aimed to analyze CAIX as a biological marker to predict RCC patient prognosis.

**Methods:**

A literature search of the PubMed and Web of Knowledge databases was performed to retrieve original studies from their inception to December of 2013. Fifteen studies, collectively including a total of 2611 patients with renal cell carcinoma, were carefully reviewed. Standard meta-analysis methods were applied to evaluate the prognostic impact of CAIX expression on patient prognosis. The hazard ratio (HR) and its 95% confidence interval (CI) were recorded for the relationship between CAIX expression and survival, and the data were analyzed using Review Manager 5.2 software and Stata software 11.0.

**Results:**

In patients with RCC, low CAIX expression was associated with poor disease-specific survival (HR = 1.89, 95% CI: 1.20–2.98, *P* = 0.006), unfavorable progression-free survival (HR = 2.62, 95% CI: 1.14–6.05, *P* = 0.02) and worse overall survival (HR = 2.03, 95% CI: 1.28–3.21, *P* = 0.002). Furthermore, low CAIX expression was significantly associated with the presence of lymph node metastases (odds ratio (OR) = 0.31, 95% CI = 0.15–0.62, *P* = 0.0009) and distant metastases (OR = 0.66, 95% CI = 0.46–0.96, *P* = 0.03) and predicted a higher tumor grade (OR = 0.41, 95% CI = 0.31–0.54, *P*<0.00001).

**Conclusions:**

Low CAIX expression most likely indicates poor prognosis in RCC patients. Moreover, low CAIX expression was significantly associated with unfavorable clinicopathological factors. To strengthen our findings, further well-designed prospective studies should be conducted to investigate the role of CAIX expression in RCC.

## Introduction

Renal cell carcinoma (RCC) is one of the most common solid cancers in humans, and its incidence is increasing annually worldwide [1]. It has been estimated that 63,920 new cases of RCC and 13,860 deaths attributable to the disease will be reported in the United States in 2014 [2]. Currently, tumor node metastasis (TNM) stage and grade are widely used to determine the prognosis of cancer, with a more advanced TNM stage related to a poorer survival [3]. There is a lack of useful biomarkers for the diagnosis and prognosis of RCC [4]. Since knowledge on the molecular mechanisms underlying tumor biology has increased, the search for prognostic biomarkers is gaining momentum. Furthermore, the identification of tissue-based RCC biomarkers that provide further prognostic information is vital for monitoring disease progression and response to therapy [3]. Consequently, a variety of biomarkers have been investigated in regards to renal carcinoma [5], [6], [7], [8], [9], [10]. Carbonic anhydrase IX (CAIX) has been considered as a candidate prognostic factor in RCC, however the supporting evidence is conflicting.

Carbonic anhydrase IX, also named MN protein, was first identified in the human cervical carcinoma cell line HeLa in 1992 [11]. CAIX expression is mediated by the hypoxia inducible factor (HIF) transcriptional complex in aberrant oxygen statuses and acidic conditions [12]. CAIX has a critical role in cancer development and progression [13]. Growing evidence indicates that CAIX is expressed at a high level in most RCC tissues and is absent in corresponding normal kidney tissues [14]. CAIX has been identified as a possible immunohistochemical predictor of RCC patient outcome [15], [16]. The association of high CAIX expression with good prognosis in patients with RCC is supported by a variety of reports. In addition, high CAIX expression has been shown to be correlated with a higher objective response rates in IL-2-treated patients [17].

Recent large cohort studies with long-term follow up found that CAIX is not an independent prognostic marker for renal carcinoma [18], [19].

In light of these conflicting results, it is of great value to investigate whether current evidence supports the use of CAIX as a prognostic marker in RCC. Therefore, this meta-analysis was conducted to elucidate the prognostic value of CAIX expression in RCC.

## Materials and Methods

### Literature search, eligibility criteria and data extraction

A literature search of original articles concerning the prognostic role of CAIX in RCC was conducted on the Pubmed and Web of Knowledge databases. The search strategy included the following keywords: renal or kidney, cancer or carcinoma, “Carbonic anhydrase IX”, CAIX or CA9, and prognosis or survival. Original articles published until December, 2013 were included in the search. The following inclusion criteria were used for the analysis: study reported in English; diagnosis of renal cell carcinoma confirmed by histopathological methods; and CAIX level detected by immunohistochemistry (IHC) analysis of primary RCC tissue. There was no exclusion criterion for the number of patients in any single study. When multiple papers were reported by the same group based on a similar patient cohort, the report with the largest number of patients was included in this study.

Data were extracted by two investigators (HZZ and GXL) independently. The extraction data included the basic information of the study (first author, publication year, country, case number), basic tumor characteristics (tumor stage and grade), cut-off value, and survival outcome (CAIX expression-related survival). Any disagreement was discussed among the author group and a consensus was made.

### Quality assessment

A quality assessment of each of the included studies was performed by two independent reviewers (HZZ and GXL) using the Newcastle-Ottawa Quality Assessment Scale for cohort studies (available at: http://www.ohri.ca/programs/clinical_epidemiology/oxford.asp) (**Table S1**). Briefly, the quality of the studies assessment included three main categories as follows: (1) selection of cohort, (2) comparability of cohort, and (3) ascertainment of outcome. This scale consisted of eight-item instrument. A study is performed by awarding 1 star for high quality elements within the selection and outcome categories. In regards to comparability, it can be awarded a maximum of 2 stars. The total number of stars is then added up and a study with more stars is reflecting a better methodological quality. Any discrepancies were resolved by discussion among the author group.

### Sensitivity analysis and publication bias

A sensitivity analysis was performed to ensure the reliability of the results [20]. In addition, funnel plots, Egger’s test and the Begg’s test were used to assess the risk of publication bias [21].

### Statistical analysis

Hazard ratios (HRs) with 95% confidence intervals (CIs) were applied to describe the impact of CAIX expression on survival. When HRs and 95% CIs were provided within the included studies, these data were extracted directly for analysis. Otherwise, the Engauge Digitizer software (version 4.1) was applied to estimate the HRs and 95% CIs from the Kaplan-Meier survival curves, and the survival rate was extracted from the curves to reconstruct the HR and its standard error (SE) [22]. In addition, odds ratios (OR) were used for the pooled analysis of the relationship between CAIX expression and clinicopathological features. The data analyses were performed with Review Manager software version 5.2 and Stata software 11.0. In addition, χ^2^ tests and I^2^ metrics were applied to estimate the heterogeneity of the studies. A random-effects model was used when the data being analyzed had significant heterogeneity [16]. A fix-effects model was used when no heterogeneity amongst the studies. All statistical tests were 2 sided, and differences were considered significant when P<0.05.

## Results

### Study Selection and Characteristics

**Figure 1:**
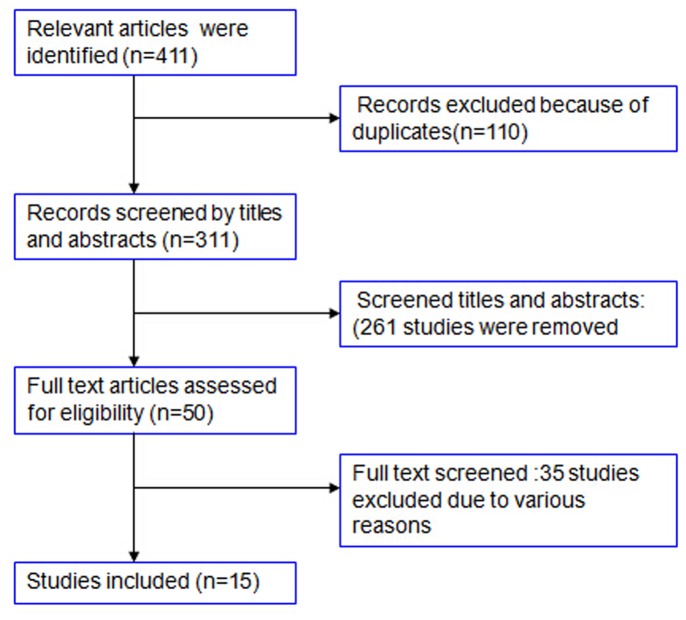
Flow chart of study selection.

From the PubMed and Web of Knowledge electronic databases, 145 and 286 potentially relevant articles were identified, respectively. In total, 421 articles were obtained through our initial search. Using Endnote software, 110 duplicated papers were excluded. A screen of the titles and abstracts identified 50 papers eligible for the assessment of the prognostic value of CAIX status in patients with RCC. After carefully review of each of the 50 studies, certain studies were excluded for the following rationale: six studies evaluated CAIX status by enzyme-linked immunosorbent assay (ELISA) [23], [24], [25], [26], [27], [28]; five studies evaluated CAIX status by real-time-PCR [29], [30], [31], [32], [33]; one study was on the topic of a single nucleotide polymorphism in the CAIX gene [34]; fourteen studies did not report survival outcome on CAIX expression or survival outcome could not be extracted [17], [35], [36], [37], [38], [39], [40], [41], [42], [43], [44], [45], [46], [47]; and nine studies contained overlapping data with other studies by the same authors or institutions [48], [49], [50], [51], [52], [53], [54], [55], [56]. Thus, fifteen papers were included in our meta-analysis to evaluate the relationship between CAIX expression and prognosis in patients with renal cell carcinoma [16], [18], [19], [57], [58], [59], [60], [61], [62], [63], [64], [65], [66], [67], [68]. The selection process is shown in **Figure 1**.

The 15 studies analyzed were conducted in: USA (x6), Korea (x1), Spain (x1),UK (x1),Germany (x1),Brazil(x1), Japan (x1), Sweden (x1), Turkey (x1) and one study included cases from both the USA and France. Our meta-analysis included a total of 2,611 patients, with a median number of 112 patients per study (range: 42–730). Of the 15 studies, six were evaluated using multivariate analysis and nine (60%) were evaluated using univariate analysis. Disease specific survival (DSS) was reported in six studies, overall survival (OS) was evaluated in seven studies and progression-free survival (PFS) was reported in five studies. The clinical characteristics of the included studies and the quality assessment are shown in **Table 1**. The details of quality assessment results of each included studies were listed in **Table S2**.

**Table 1:** Basic characteristics of included studies and quality assessment.

Firstauthor	Year	Country	Cases	T stage	Grade	Cut-offvalue	Survivalout come	Survivalanalysis	Quality assessment
Atkins M	2005	USA	66	NA	NA	85%	OS	Univariate	6
Biswas S	2012	UK	112	NA	NA	NA	OS	Multivariate	7
Bui MH	2003	USA	321	114/39/150/18	38/151/110/22	85%	DSS	Univariate	7
Choueiri TK	2012	USA	133	NA	NA	85%	PFS	Univariate	8
Dornbusch J	2013	Germany	42	10/3/27/2	1/20/12/9	NA	OS, PFS	Multivariate	7
Dudek AZ	2010	USA	47	NA	NA	85%	OS,PFS	Univariate	5
Kim HS	2011	Korea	56	NA	16/39/6/1	85%	PFS	Univariate	7
Klatte T	2007	USA	357	117/36/141/14	41/152/106/9	NA	DSS	Multivariate	6
Muriel LC	2012	Spain	135	NA	NA	85%	PFS,OS	Univariate	6
Patard JJ	2005	France and USA	100	29/20/48/3	2/37/41/20	85%	DSS	Multivariate	7
Phuoc NB	2008	Japan	122	57/18/17/30	27/71/20/4	Score 4	DSS	Multivariate	6
Sandlund J	2007	Sweden	228	NA	NA	10%	DSS	Multivariate	7
Soyupak B	2005	Turkey	67	22/31/8/6	24/20/16/7	50%	OS	Univariate	7
Zerati M	2013	Brazil	95	69/8/18/0	25/37/26/7	NA	OS	Univariate	6
Zhang BY	2013	USA	730	NA	NA	85%	DSS	Univariate	7

Abbreviation: NA, not available; OS, overall survival; DSS, disease-specific survival; PFS, progression-free survival.

**Figure 2:**
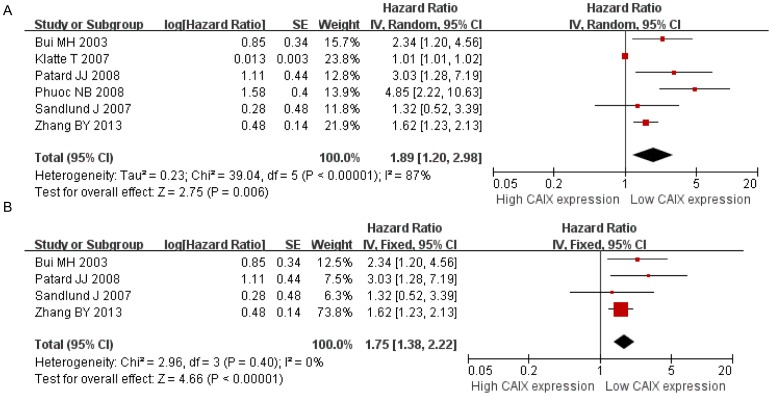
Meta-analysis of CAIX expression and disease-specific survival on A, all inclusion studies; B, by excluding the low quality score studies (quality score≤6).

We aimed to determine whether CAIX expression levels were associated with the survival of patients with RCC. Six of the studies included were used to evaluate the association of low CAIX expression levels with DSS. The combined HR of the studies revealed that low CAIX expression levels were associated with poorer DSS in patients with RCC (HR = 1.89, 95% CI: 1.20–2.98, *P* = 0.006, **Figure 2A**). The difference in the heterogeneity of the data between studies was significant (Chi^2^ = 39.04; *P*<0.00001, I^2^ = 87%). In addition, a sensitivity analysis was performed. The omission of any one study did not significantly influence the results. On the premise of these stability results, there is no doubt that CAIX expression is a prognostic marker for DSS in patients with renal cell carcinoma. Moreover, subgroup analysis was performed by excluding the low quality studies (quality score≤6), and the result showed that decreased CAIX expression was also associated with poorer DSS (HR = 1.75, 95% CI: 1.38–2.22, *P*<0.00001). The analysis had no heterogeneity (I^2^ = 0, *P* = 0.40), and the results were showed in **Figure 2B** **.**

**Figure 3:**
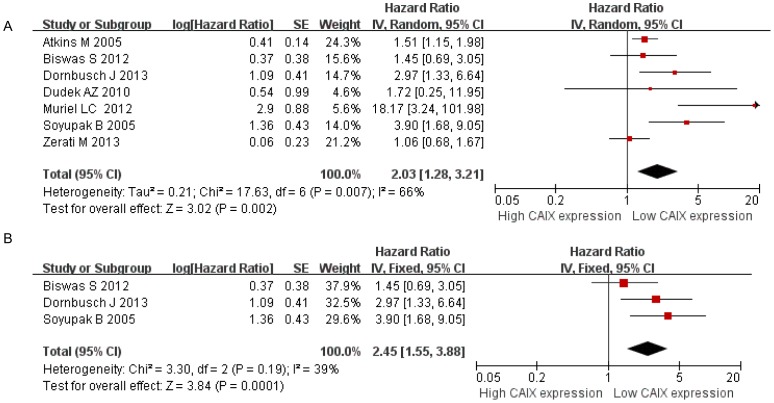
Meta-analysis of CAIX expression and overall survival on A, all inclusion studies; B, by excluding the low quality score studies (quality score≤6).

Seven studies were included to evaluate the correlation between low CAIX expression and OS. The results indicated that low CAIX expression predicted an unfavorable OS (HR = 2.03, 95% CI: 1.28–3.21, *P* = 0.002, **Figure 3A**); however, the heterogeneity between the studies was significant (Chi^2^ = 17.63; *P* = 0.007, I^2^ = 66%). Sensitivity analysis was conducted by sequential omission of any single study, the results showed low CAIX expression was correlated with unfavorable OS. Moreover, subgroup analysis was conducted by excluding the study with low quality (quality score≤6), the low CAIX expression was also associated with poorer OS (HR = 2.45, 95% CI: 1.55–3.88, *P* = 0.0001), and there was no significant heterogeneity (I^2^ = 39%, *P* = 0.19, **Figure 3B**).

**Figure 4:**
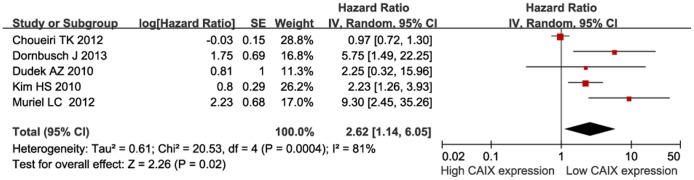
Meta-analysis of CAIX expression and progression-free survival.

**Figure 5:**
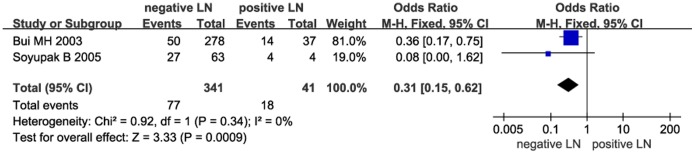
Meta-analysis of the association between low CAIX expression and lymph node metastasis.

**Figure 6:**
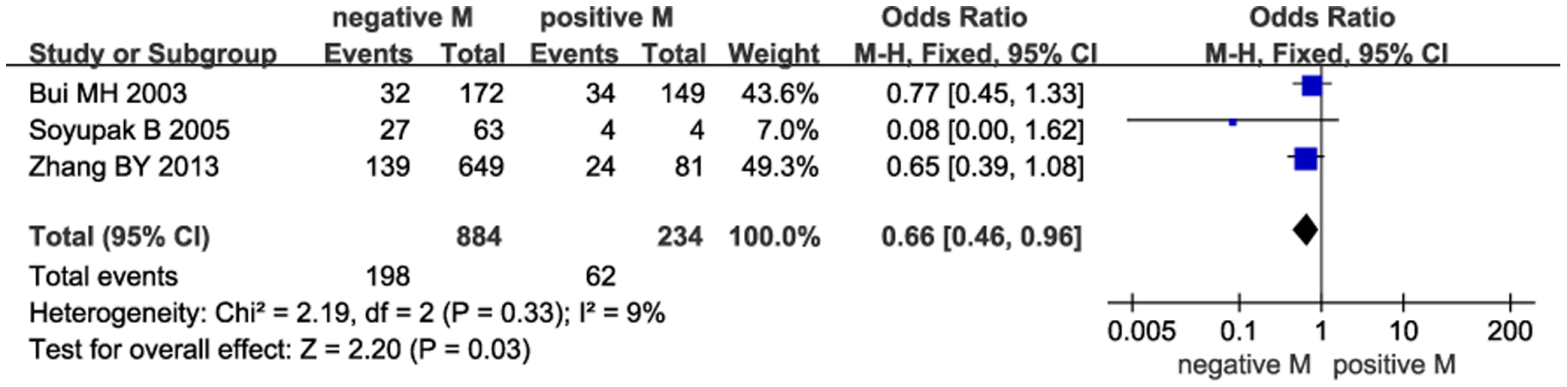
Meta-analysis of the association between low CAIX expression and distant metastasis.

**Figure 7:**
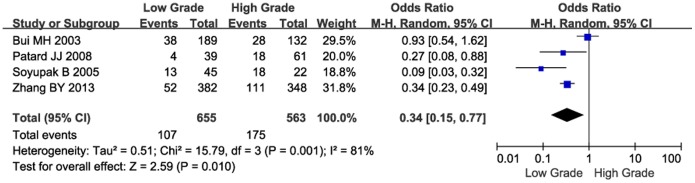
Meta-analysis of the association between low CAIX expression and grade.

**Figure 8:**
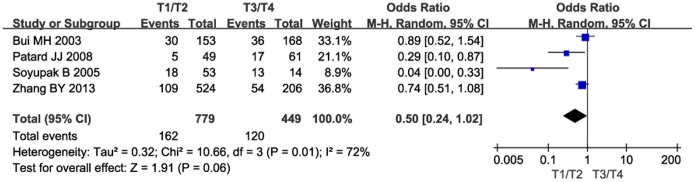
Meta-analysis of the association between low CAIX expression and depth of invasion.

Next, we investigated the relationship between CAIX expression and PFS. The pooled analysis of the studies reporting PFS outcomes indicated that low CAIX expression might predict poor PFS (HR = 2.62, 95% CI: 1.14–6.05, *P* = 0.02, **Figure 4**). Furthermore, we investigated the correlation between low CAIX expression and clinicopathological features. The results were listed in **Table 2**. Our study revealed that low CAIX expression was associated with the presence of lymph node metastases (OR = 0.31, 95% CI = 0.15–0.62, *P* = 0.0009, **Figure 5**) and distant metastases (OR = 0.66, 95% CI = 0.46–0.96, *P* = 0.03, **Figure 6**). None of these analyses showed significant heterogeneity between the studies. This meta-analysis also showed that low CAIX expression was significantly correlated with worse RCC grade (OR = 0.41, 95% CI = 0.31–0.54, *P*<0.00001, **Figure 7**) and depth of invasion (OR = 0.50, 95% CI = 0.24–1.02, *P* = 0.06, **Figure 8**).

**Table 2:** Meta-analysis assessing the relationship between low CAIX expression and clinicopathological variables.

Clinicopathological variable	No.of studies	No.of patients	Pooled HR^a^			Heterogeneity	
			HR	95%CI	*P* value	I^2^(%)	*P* value
Lymph Node Metastasis(negative/positive)	2	382	0.31	0.15–0.62	0.0009	0	0.34
Distant metastasis(negative/positive)	3	1118	0.66	0.46–0.96	0.03	9	0.33
Grade (low/high)	4	1218	0.34	0.15–0.77	0.01	81	0.001
Depth of invasion (T1+2/T3+4)	4	1228	0.50	0.24–1.02	0.06	72	0.01

a if I^2^≤50%, a fix-effects model was used, if I^2^>50%, a random-effects model was used. HR, hazard ratio; CI, confidence interval.

**Figure 9:**
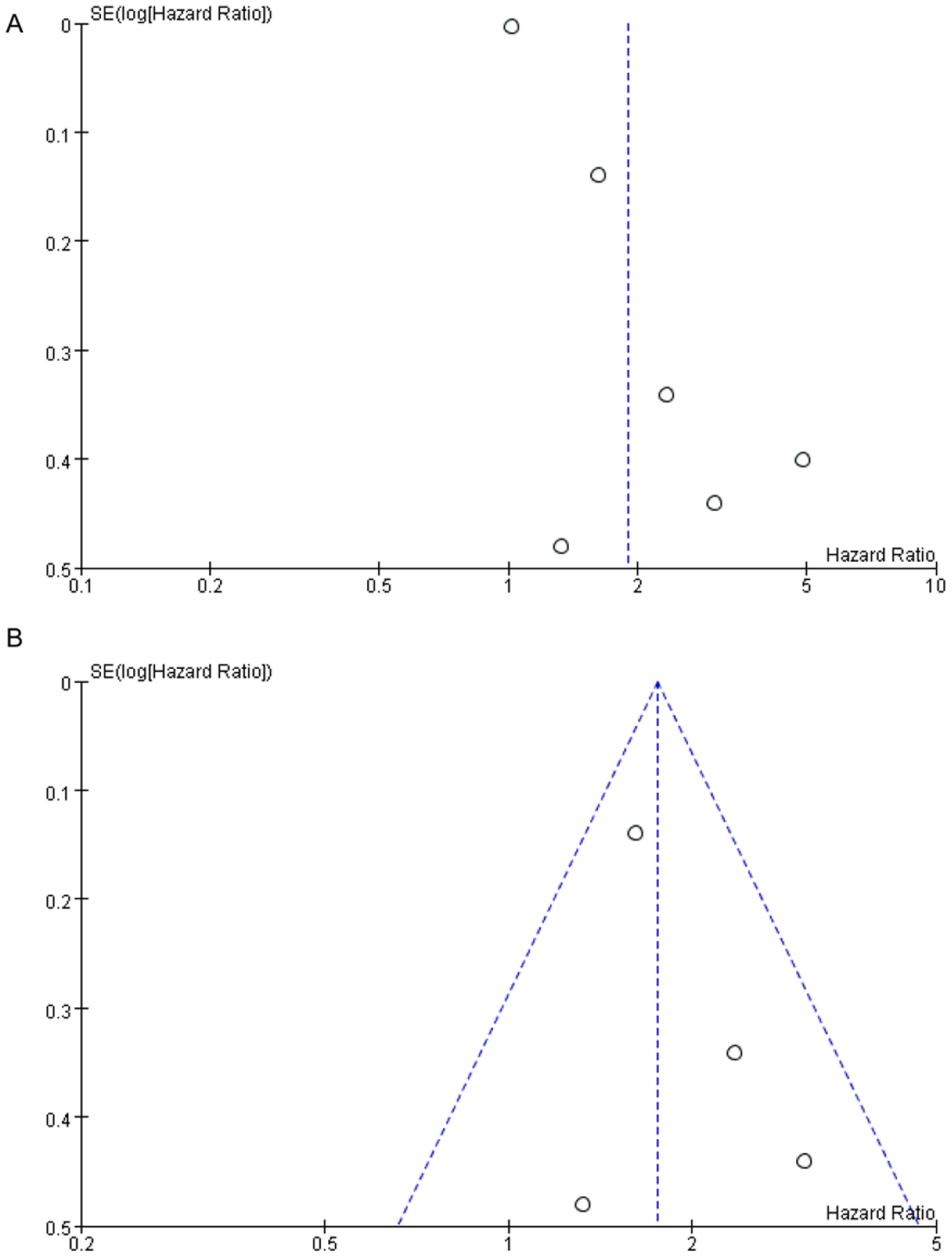
Funnel plot of CAIX expression and disease-specific survival on A, all inclusion studies; B, by excluding the low quality score studies (quality score≤6). SE = standard error, HR = hazard ratio.

**Figure 10:**
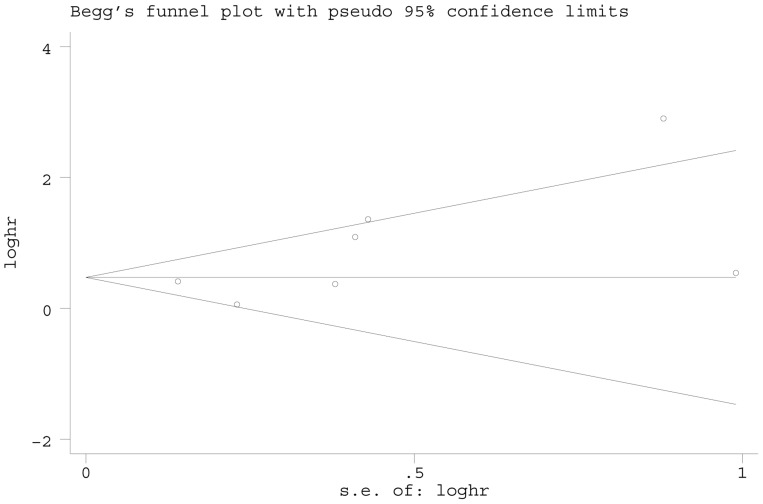
Begg’s funnel plot of CAIX expression and overall survival. SE = standard error, HR = hazard ratio.

Publication bias was assessed for DSS and OS, respectively. For DSS, the funnel plot of HR indicated some degree of publication bias (**Figure 9A** **)**. However, by excluded the study with low quality, there was no significant publication bias by statistical test (Egger’s test = 0.451, Begg’s test = 0.734, **Figure 9B**). For OS, the funnel plot of the HR showed no obvious publication bias (**Figure 10**), and the *P* value of statistical test (Egger’s test = 0.123, Begg’s test = 0.072) indicated no significant publication bias.

## Discussion

Prognostic marker can indicate the course of a disease and have multiple applications in disease diagnosis, treatment and the prediction of clinical outcome.

Numerous studies have evaluated CAIX as a prognostic marker for RCC with conflicting results. Thus, this meta-analysis aimed to clarify the prognostic role of CAIX in RCC; the results suggest that low CAIX expression is associated with poor DSS (HR  = 1.89, 95% CI: 1.20–2.98, *P* = 0.006), worse OS (HR = 2.03, 95% CI: 1.28–3.21, *P* = 0.002) and unfavorable PFS (HR = 2.62, 95% CI: 1.14–6.05, *P* = 0.02). These results indicate the potential of CAIX as a valuable biological marker to predict prognosis in patients with RCC. Interestingly, CAIX has been shown to be an effective biological marker for determining the best course of treatment for specific patients with RCC and is currently under further investigation [23], [69]. Several studies have also suggested a positive correlation between CAIX levels and the IL-2 response of patients with RCC undergoing treatment [57], [59], [61]. However, a significant association has not been demonstrated between CAIX expression and clinical outcome in patients treated with sorafenib or temsirolimus [70]. We also investigated the association between CAIX expression and RCC clinical characteristics. Interestingly, low CAIX expression was correlated with a number of characteristics including: high RCC grade, the existence of lymph node metastases and distant metastases. Furthermore, low CAIX expression was related to the depth of invasion, although the *P* value did not reach the level of statistical significance (*P* = 0.06). Some reports have shown that low CAIX expression was associated with a more aggressive subtype in RCC [30], [59]. Another study showed that CAIX was strongly associated with vascular invasion in RCC [29]. We did not evaluate the association between CAIX expression and TNM stage since only one study reported a significant correlation between low CAIX level and TNM stage [25].

However, in contrast to the results of the studies on RCC, certain studies have indicated that high CAIX expression predicts poor prognosis in patients with other cancers, such as ovarian, gastric, lung, etc. [71], [72], [73]. The mechanism for this difference is unclear, however one possible explanation relates to aberrations in VHL, which have been detected in the majority of RCCs [65]. Thus, VHL tumor suppressor gene inactivation rather than HIF activation may be the cause of high CAIX expression in patients with RCC [74].

A previous study showed that VHL mutational status is significantly associated with high CAIX expression [65]. Additional studies, therefore are required to determine the mechanism of the prognostic role of CAIX in patients with RCC.

The following limitations of this meta-analysis should be considered. Firstly, the studies included in this meta-analysis were limited to those published in the English language because the authors of this current study were not literate in other languages. Thus, studies published in English may have more frequently supported our hypotheses, and studies reported in other languages may have more frequently refuted our hypotheses [75]. Another possible bias was that the level of evidence provided by observational studies was less than that provided by randomized controlled trials. Most of the studies included in our meta-analysis were retrospective studies, and only one randomized clinical trial was available [16]. Secondly, it is important to evaluate the value of a prognostic marker based on the results of randomized clinical trials; however, due to the limited number of randomized clinical trials in our meta-analysis, the prognostic role of CAIX expression level in RCC should be interpreted with caution.

In our study, there was significant heterogeneity among the 15 included studies. Heterogeneity could have been caused by the following factors: individual patients coming from different countries with different histological types and tumor stages, the therapy methods used, cut-off values, different sources and dilutions of primary antibodies, follow-up times and other factors. To minimize heterogeneity, the association between CAIX expression and prognosis was evaluated based on different survival outcomes (DSS, OS and PFS), and only studies that measured CAIX expression levels with immunohistochemistry were included. Studies that measured CAIX expression levels using ELISA or real time-PCR were not included in our analysis. However, subgroup analysis was performed by excluding the studies with low quality scores, there was no significant heterogeneity for DSS and OS, respectively (**Figure 2B** **and** **Figure 3B**).

Another limitation to our study was the process of data extraction. For studies that did not provide HR and SE directly, the data was calculated by using survival curves. This process introduced a potential source of bias. The estimated HRs and SEs may have been less accurate than those provided directly by the studies and those calculated from the data reported by the studies.

Moreover, we should aware the publication bias issue. For DSS, the results indicated existing publication bias. However, when excluded the study with low quality score, no publication bias was detected (**Figure 9B**). Furthermore, there was no significant publication bias for OS (**Figure 10**), which indicated the analyses are feasible and the results are credible.

In conclusion, this study is the first meta-analysis to comprehensively and systematically evaluate the association of CAIX expression with the survival and clinical characteristics of patients with RCC. Our meta-analysis indicated that low CAIX expression detected by IHC was associated with poor DSS and OS in patients with RCC. Furthermore, low CAIX expression was correlated with lymph node metastases, distant metastases and high RCC grade. To further strengthen our findings, well-designed prospective studies with more standardized assessments of prognostic markers should be performed.

## Supporting Information

Table S1 **Newcastle – Ottawa quality assessment scale.**
(DOCX)

Table S2 **Quality assessment of each study included.**
(DOCX)

Checklist S1 **PRISMA checklist for this meta-analysis.**
(DOC)

Data S1 **Raw data and the final data for survival outcome.**
(DOCX)
