# Supplementary material for: Prognostic Value of Carbonic Anhydrase IX Immunohistochemical Expression in Renal Cell Carcinoma: A Meta-Analysis of the Literature
Source: PLoS One. 2014 Nov 26;9(11):e114096. doi: 10.1371/journal.pone.0114096 (PMC4245260; doi:10.1371/journal.pone.0114096)
Supplement: Table S1 — Newcastle – Ottawa quality assessment scale. (DOCX) [file pone.0114096.s001.docx]

Table S1 Newcastle – Ottawa quality assessment scale

| Item |
| --- |
| Selection |
| (1) Representativeness of the exposed cohort  (a) Truly representative of the average ‘renal cell carcinoma patient’ in the community (1 star)  (b) Somewhat representative of the average ‘renal cell carcinoma patient’ in the community  (1 star)  (c) Selected group of users (e.g. nurses, volunteers)  (d) No description of the derivation of the cohort |
| (2) Selection of the non-exposed cohort  (a) Drawn from the same community as the exposed cohort (1 star)  (b) Drawn from a different source  (c) No description of the derivation of the non-exposed cohort |
| (3) Ascertainment of exposure (Proof of renal cell carcinoma and CAIX measurement)  (a) Secure record (eg surgical records) (1 star)  (b) Structured interview (1 star)  (c) Written self-report  (d) No description |
| (4) Demonstration that outcome of interest was not present at start of study  (a) Yes (1 star)  (b) No |
| Comparability |
| (1) Comparability of cohorts on the basis of the design or analysis  (a) Study controls for ‘metastasis or recurrence’ (1 star)  (b) Study controls for any additional factor (1 star) (Age, stage, grade etc.) |
| Outcome |
| (1) Assessment of outcome (Death or recurrence)  (a) Independent blind assessment (1 star)  (b) Record linkage (1 star)  (c) Self-report  (d) No description |
| (2) Was follow-up long enough for outcomes to occur? (Death or recurrence)  (a) Yes (‘3 years’) (1 star)  (b) No |
| (3) Adequacy of follow-up of cohorts  (a) Complete follow-up – all subjects accounted for (1 star)  (b) Subjects lost to follow-up unlikely to introduce bias – small number lost  ‘(25%)’ or description provided of those lost (1 star)  (c) Follow-up rate ‘<75%’ and no description of those lost  (d) No statement |
