# Supplementary material for: Prognostic Value of Carbonic Anhydrase IX Immunohistochemical Expression in Renal Cell Carcinoma: A Meta-Analysis of the Literature
Source: PLoS One. 2014 Nov 26;9(11):e114096. doi: 10.1371/journal.pone.0114096 (PMC4245260; doi:10.1371/journal.pone.0114096)
Supplement: Table S2 — Quality assessment of each study included. (DOCX) [file pone.0114096.s002.docx]

Table S2 Quality assessment of each study included

| Study | Selection | | | | Comparability | Outcome | | | Quality assessment star |
| --- | --- | --- | --- | --- | --- | --- | --- | --- | --- |
|  | (1) | (2) | (3) | (4) | （1） | (1) | (2) | (3) |  |
| Atkins M | b* | a* | a* | b | b* | b* | a* | d | 6* |
| Biswas S | b* | a* | a* | a* | a* | b* | a* | d | 7* |
| Bui MH | b* | a* | a* | b | b* | b* | a* | a* | 7* |
| Choueiri TK | b* | a* | a* | b | a*b* | b* | a* | a* | 8* |
| Dornbusch J | c | a* | a* | b | a*b* | b* | a* | a* | 7* |
| Dudek AZ | c | a* | a* | b | b* | b* | a* | d | 5* |
| Kim HS | c | a* | a* | b | a*b* | b* | a* | a* | 7* |
| Klatte T | b* | a* | a* | b | b* | b* | a* | d | 6* |
| Muriel LC | c | a* | a* | b | b* | b* | a* | a* | 6* |
| Patard J J | b* | a* | a* | b | a*b* | b* | a* | d | 7* |
| Phuoc NB | c | a* | a* | b | b* | b* | a* | a* | 6* |
| Sandlund J | b* | a* | a* | b | b* | b* | a* | a* | 7* |
| Soyupak B | b* | a* | a* | b | b* | b* | a* | b* | 7* |
| Zerati M | c | a* | a* | b | b* | b* | a* | a* | 6* |
| Zhang BY | b* | a* | a* | b | b* | b* | a* | a* | 7* |
