# Supplementary material for: Prognostic Value of Carbonic Anhydrase IX Immunohistochemical Expression in Renal Cell Carcinoma: A Meta-Analysis of the Literature
Source: PLoS One. 2014 Nov 26;9(11):e114096. doi: 10.1371/journal.pone.0114096 (PMC4245260; doi:10.1371/journal.pone.0114096)
Supplement: Data S1 — Raw data and the final data for survival outcome. (DOCX) [file pone.0114096.s004.docx]

1.Data extraction for disease-specific survival

| First author | year | Survival  analysis | survival data extraction | out come | HR | 95%CI |
| --- | --- | --- | --- | --- | --- | --- |
| Bui MH | 2004 | Univariate | curves | DSS | 1.54 | 0.62- 3.79 |
| Klatte T | 2007 | Multivariate | reported | DSS | 0.987^a^ | 0.981-0.993 |
| Patard J J | 2005 | Multivariate | reported | DSS | 0.33^a^ | 0.14-0.79 |
| Phuoc NB | 2008 | Multivariate | reported | DSS | 0.33^a^ | 0.172-0.634 |
| Sandlund J | 2007 | Multivariate | calculated | DSS | 1.32 | 0.60-2.93 |
| Zhang BY | 2013 | Univariate | reported | DSS | 1.62 | 1.24-2.11 |

a indicated high caix expression versus low caix expression HR,hazard ration CI: confidence interval

2. Data extraction for overall survival

| First author | year | Grade（I/II/II/IV） | Survival  analysis | survival data extraction | Survival out come | HR | 95%CI |
| --- | --- | --- | --- | --- | --- | --- | --- |
| Atkins M | 2005 | NA | Univariate | curves | OS | 1.51 | 1.15-1.98 |
| Biswas S | 2012 | NA | Multivariate | reported | OS | 1.45 | 0.69-3.03 |
| Dornbusch J | 2013 | 1/20/12/9 | Univariate | reported | OS | 0.335 | 0.151-0.742 |
| Dudek AZ | 2010 | NA | Univariate | Calculated | OS | 1.72 | 0.25-11.95 |
| Muriel LC | 2012 | NA | Univariate | Calculated | OS | 18.17 | 3.24-101.98 |
| Soyupak B | 2005 | 24/20/16/7 | Univariate | reported | OS | 3.90 | 1.68-9.05 |
| Zerati M | 2013 | NA | Univariate | curvers | OS | 1.06 | 0.68-1.67 |

HR,hazard ration CI: confidence interval

3.Data extraction for progression-free survival

| First author | year | Survival  analysis | method for survival data extraction | Survival out come | HR | 95%CI |
| --- | --- | --- | --- | --- | --- | --- |
| Choueiri TK | 2012 | Univariate | calculated | PFS | 0.97 | 0.72-1.30 |
| Dornbusch J | 2013 | Univariate | reported | PFS | 0.559 | 0.294-1.062 |
| Dudek AZ | 201 | Univariate | calculated | PFS | 2．25 | 0．32-15．96 |
| Kim HS | 2011 | Univariate | calculated | PFS | 2.23 | 1.26-3.93 |
| Muriel LC | 2012 | Univariate | calculated | PFS | 9.30 | 2.45-35.26 |
